# Is there a crisis in nursing retention in New South Wales?

**DOI:** 10.1186/1743-8462-5-19

**Published:** 2008-08-05

**Authors:** Denise Doiron, Jane Hall, Glenn Jones

**Affiliations:** 1 School of Economics, University of New South Wales, Sydney, Australia & Research Associate CHERE, University of Technology, Sydney, Australia; 2 Centre for Health Economics Research and Evaluation, University of Technology, Sydney, Australia; 3 School of Economic and Financial Studies, Macquarie University, Sydney Australia & Research Associate, CHERE, University of Technology, Sydney, Australia

## Abstract

**Background:**

There is a severe shortage of nurses in Australia. Policy makers and researchers are especially concerned that retention levels of nurses in the health workforce have worsened over the last decade. There are also concerns that rapidly growing private sector hospitals are attracting qualified nurses away from the public sector. To date no systematic analysis of trends in nursing retention rates over time has been conducted due to the lack of consistent panel data.

**Results:**

A 1.4 percentage point improvement in retention has led to a 10% increase in the overall supply of nurses in NSW. There has also been a substantial aging of the workforce, due to greater retention and an increase in mature age entrants. The improvement in retention is found in all types of premises and is largest in nursing homes. There is a substantial amount of year to year movement in and out of the workforce and across premises. The shortage of nurses in public hospitals is due to a slowdown in entry rather than competition from the rapidly growing private sector hospitals.

**Policy Implications:**

The finding of an improvement (rather than a worsening) in retention suggests that additional improvements may be difficult to achieve as further retention must involve individuals more and more dissatisfied with nursing relative to other opportunities. Hence policies targeting entry such as increased places in nursing programs and additional subsidies for training costs may be more effective in dealing with the workforce shortage. This is also the case for shortages in public sector hospitals as retention in nursing is found to be relatively high in this sector. However, the large amount of year to year movements across nursing jobs, especially among the younger nurses, also suggests that policies aimed at reducing job switches and increasing the number who return to nursing should also be pursued. More research is needed in understanding the relative importance of detailed working conditions and the problems associated with combining family responsibilities and nursing jobs.

## Background

There is currently a severe shortage of experienced nurses in Australia, as in many other developed countries. This is considered to be the result of both decreasing enrolments in nursing education, and poor retention rates of those in the nursing workforce. Retention rates are generally thought to have worsened over recent years. There is also concern over the ageing of the nursing workforce since this will cause critical shortages in the future as greater proportions of nurses reach retirement age.

The Senate Select Committee on Nursing described workforce retention as an acute problem, and a key issue in ensuring adequate numbers in the future. The reasons given for nurses leaving the workforce include pay and conditions, increased workload, particularly in acute hospitals with higher complexity patients admitted for shorter stays, lack of childcare, and poor recognition of nurses' skills and knowledge [1]. There is a general perception that, as workloads have increased, nurses feel undervalued and increasingly stressed, retention rates fall further increasing workloads, leading to higher levels of stress and burnout, and a further decrease in retention. However, there is no readily available information on the number of nurses leaving the workforce.

The Australian Institute of Health and Welfare reports the number of nurses employed in nursing, for all nurses and registered and enrolled nurses separately [2]. While this provides some indication of the size of a potential pool of trained nurses, it does not show retention rates, their changes over time, or whether they vary according to the type of job, the type of hospital, or type of nurse. A report by the National Nursing and Nursing Education Taskforce shows that nursing students are more likely to complete their course and to find employment in their field than other higher education programs. However, no data are available on attrition once in employment, and, as stated in the report, "there is little good data that allows us to know what is actually occurring within nursing or to compare nursing to other careers" [[3], p2].

The issue of the public and private sector distribution of resources is of great interest to policy makers and to researchers. The growth in private hospitals over the last twenty years raises the possibility that the shortage of nurses in the public sector is, in large part, due to a shift of resources to the rapidly growing area of private health care. Understanding the allocation of the nursing workforce in the two sectors, and changes over time in this allocation is important in developing policies towards solving the nursing shortage.

There is very little work in the economics literature on the nursing workforce in Australia. The reason for this is again the lack of appropriate data. Shah and Long use data from the Labour Force Survey (LFS) to predict the supply of nurses and other health care workers as well as shortages in these professions based on a forecast of the demand for health care [4]. Given the small sample of nurses in general surveys such as the LFS, the potential for detailed micro-econometric analysis based on these data is limited. In an earlier article, Doiron and Jones use NSW administrative data to analyze the relationship between hospital characteristics and nurse retention in 1996–97. Based on cross-section variation in the characteristics of nurses and the hospitals in which they work, this study finds that several hospital characteristics significantly affect the probability of retention. Characteristics which positively affect retention include hospital size, expenditures, emergency admissions and staffing levels. Factors which negatively affect retention include workloads, complexity (ANDRG weight), and greater usage of visiting medical officers. Surprisingly, no evidence of hospital specific effects over and above observed hospital characteristics was found [5].

Nursing shortages are a concern in many countries and a significant number of studies on nursing labour supply have been conducted in recent years based on overseas data. Reviews of the literature are provided in Shields [6], and Antonazzo, Scott, Skatun and Elliott [7].

A larger body of work on the nursing shortage in Australia can be found in nursing journals and government reports [8-11]. Many of the issues and concerns over the present state of the nursing profession discussed above are drawn from these studies. The current article contributes to the area by providing more quantitative and evidence-based results on nursing retention over time. Specifically, we use administrative data on New South Wales (NSW) nurses to analyze trends over the period 1993–2000 in the nursing workforce: the aging of the population of workers, the attrition rates by age group and by premises, and the year to year movements across nursing jobs as well as in and out of nursing.

## Methods

### Data

All working nurses in NSW must be registered with the NSW Nurses Registration Board. We begin by using aggregate figures based on this information and published by NSW Health. These data allow us to construct a consistent data series on the size of the nursing workforce in NSW from 1986 to 2000. This is informative in establishing trends over time in the total supply of nurses in NSW.

At the time of registration, those nurses renewing their registration (rather than registering for the first time) are given a labour force questionnaire which covers personal characteristics and, if working in NSW, information about the job including location, hours of work, and type of work. Unit record files based on the information collected by the NSW Nurses Registration Board from the registration renewal form and the labour force questionnaire have been provided to us in a de-identified form by the Australian Institute of Health and Welfare (AIHW) with NSW Health's permission. Each nurse is given a unique identifier (a random number unrelated to the nurses' registration number) permitting the linkage of individuals over time. The data span the years 1993–2000, excluding 1998.

Estimates of the response rate to the labour force questionnaire suggest that the data represent between 85 and 90% of the total nursing workforce in NSW [2]. Hence we can follow the working history of a large sample of nurses over a fairly long panel. With these data, we can analyse retention and changes in retention by type of premises and nurse characteristic. We can also look at nurses who remain in nursing but who change type of premises from year to year. These data form the basis of all quantitative analyses reported below.

### Analysis

Retention rates are calculated separately for different types of nurses and jobs, and for different time periods. To further quantify changes over time, separate retention probit regressions are estimated for 1994–95 and 1999–2000. Separate probit regressions are also estimated for RNs and enrolled nurses. Explanatory variables include controls for personal characteristics and proxies for human capital (sex, age, citizenship, post basic education, years since first registered as a nurse); job characteristics (hours of work, an indicator for the presence of a second job in nursing, field, activity, job classification, location of the job, and premises); and the local unemployment rate. There is no information on wages; however most nurses will be covered by award wages and hence, their wages will be determined in large part by their job classification. This analysis is useful in allowing us to separate out and quantify the contribution of these various characteristics to retention and to changes in retention over time. Finally, cohorts of nurses are followed over time in order to describe the profile of work experience by nurses.

## Results

### General trends in the nursing workforce

**Figure 1:**
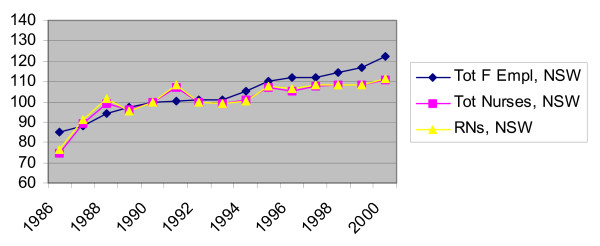
**Trends in Total Female Employment and Nursing, NSW(1990 = 100)**. Source: Profile of the Nursing Workforce, NSW Department of Health, various issues.

Figure 1 presents trends in the employment of nurses in total and RNs only in NSW from 1986 to 2000. For comparison, the trend in total female employment in the State is also plotted. Figure 1 shows that the growth rate in the total number of nurses has slowed in the 90s compared to 1986–1989. It has also slowed relative to the trend in total employment of women in the state. The trend in the employment of RNs follows closely that of the total nursing employment. RNs comprise between 80 and 85% of total nurses in the state and, despite the concerns that less skilled nurses are being increasingly used as substitutes for RNs, no definite trend is detected in the proportion of RNs to enrolled nurses over the period in question.

### Nurse retention by age

We now make use of the detailed unit record data from the NRB. Table 1 presents statistics on the average age of the workforce and the proportion of nurses in the younger age groups: less than 25 years of age and 25–29 years of age. A nurse is defined as working if he/she either a) answers the labour force question and indicates working in NSW or b) provides positive hours of work in nursing in NSW. With this definition, some nurses who are providing positive hours of unpaid work may be included as working, but it does provide consistent estimates over the period under study. More details are available from the authors.

**Table 1:** Average age and proportion of nurses in young age groups

	**Total working nurses**				**RNs only**			
		
**Year**	**Number working**	**Average age**	**% aged <25**	**% aged 25–29**	**Number working**	**Average age**	**% aged <25**	**% aged 25–29**
1993	40762	38.75	6.76	12.30	33200	39.45	6.30	11.12
1994	47946	39.35	4.99	10.66	40296	40.01	4.19	9.51
1995	50793	39.72	5.17	10.62	43116	40.29	4.44	9.85
1996	51497	40.08	4.95	10.72	44025	40.62	4.39	10.16
1997	53076	40.38	5.02	10.51	44735	40.91	4.48	10.08
1998	-	-	-	-	-	-	-	-
1999	53376	41.80	3.10	9.67	45100	42.25	2.76	9.34
2000	54648	42.16	2.86	9.63	46249	42.54	2.63	9.47

Notes: The samples exclude nurses who do not report their age; this group forms less than 5% of the sample.

The third column shows an increase in the average age of working nurses over the period from just under 39 in 1993 to over 42 in the year 2000, a substantial increase in a relatively short period of time. A similar increase in the average age is found when considering RNs only.

The next set of columns suggests that one cause of this aging is a substantial reduction in nurses in the youngest age group. The percentage of all working nurses in this age group falls from almost 7% in 1993 to less than 3% by the year 2000. There is in fact a large drop in the absolute number of both RNs and enrolled nurses less than 25 years of age from 1997 to 2000. Specifically, the number of working RNs (enrolled nurses) under 25 years of age went from 2004 (660) in 1997 to 1245 (410) in 1999 and 1216 (347) in 2000. This is the result of a decrease in the entry into nursing, bachelor degree non-overseas student commencements dropped from 11,274 in 1994 to 8,248 in 2000 [12].

The reduction in the under 25 cohort has started to feed into the next age group (25–29) and a continued drop in the numbers in this age group is expected in the next few years. Any trend to increase the proportion of mature age entrants to nursing will also affect the age profile of the workforce. Whether this will contribute to an aging of the workforce depends on the age distribution of this pool of entrants.

**Figure 2:**
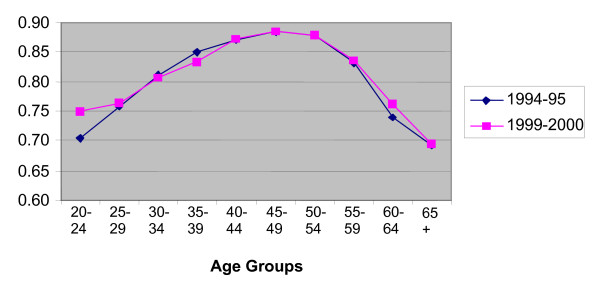
**Retention Rates by Age Group, RNs**. Source: Profile of the Nursing Workforce, NSW Department of Health, various issues.

**Figure 3:**
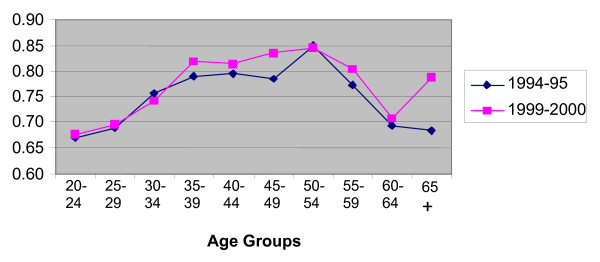
**Retention Rates by Age Group, Enrolled Nurses**. Source: Profile of the Nursing Workforce, NSW Department of Health, various issues.

In presenting evidence on retention, we focus on data for 1994–95 and 1999–2000. These years span most of the available period and the growth rates for 1994–95 and 1999–2000 in the microdata match very closely the official estimates published by NSW Health. Figures 2 and 3 plot retention rates by age group for RNs and enrolled nurses. Retention rates have been calculated as follows. For each year, all nurses working in NSW are identified and categorised by age group. Using the individual identifier, we see if the nurse is still working in NSW in the following year. If the answer is 'yes', the nurse is part of the retained group. This is true whether or not the nurse is still in the same job premises. For example, a nurse working in a public hospital in 1994 and working in a private nursing home in 1995 is considered as retained. This definition of retention is appropriate when analysing nurses' decisions to remain or leave nursing rather than the choice of staying in particular work premises.

The overall retention rate for nurses in 1994–95 is 82.3%; hence 17.7% have stopped working in nursing in NSW in 1995 at least temporarily. The rate for RNs is higher than for enrolled nurses (83.6 versus 75.6%). Retention rates are lowest for the extreme age groups. This is not unique to nursing. The young are known to have high mobility rates across jobs and labour market states, and this is usually explained by job shopping and attempts to improve on the quality of the job match. Since most nurses are female, low retention rates in the child bearing years is also expected. The old are retiring from the labour force. For nurses in NSW, the highest retention rates are found for the 40–55 age groups.

How do these rates compare with overseas nurses or with other occupations? According to Fritjers et al [12], the turnover rate among nurses in the British National Health Service is approximately 10% while McCarty et al [13] report rates for the USA between 20% and 30%. There is sparse evidence of turnover by occupation generally but numbers involving teachers suggest similar attrition. For example, Clotfelter et al calculate year on year attrition rates amongst teachers in North Carolina at around 26% [14].

The surprising fact shown in Figures 2 and 3 is that retention rates in NSW have not worsened over the period 1995–2000. On average and for most age groups, they have slightly improved. Overall, the rate increased from 82.3% to 83.3% with the largest increases found amongst the enrolled nurses and generally in the youngest age groups. These figures suggest that the aging of the nursing workforce originates from fewer entrants, particularly of school leavers, and a slight improvement in retention.

### Growth and retention by type of premises

Table 2 shows the distribution of nurses by broad categories of type of premises for 1994 and 2000. Growth rates are computed separately for public and private sectors. Overall there has been a slight increase in the proportion of all nurses working in the public sector from 72% in 1994 to 75% in 2000. The private sector overall does not show an increase in the employment of nurses over the period and the aggregate 10% rise in the nursing workforce is found in the total public sector employment growth.

**Table 2:** Growth of the NSW nursing workforce by premises

	**1994**		**2000**		**Growth 2000-1994**		
			
**Premises**	**Number working**	**Prop. private**	**Number working**	**Prop. private**	**Public 00/94**	**Private 00/94**	**Total 00/94**
**All nurses**							
hospital	30332	0.16	34816	0.18	1.11	1.33	1.15
nursing homes	8428	0.62	8485	0.45	1.46	0.73	1.01
community health	3100	0.00	4744	0.07	1.42		1.53
develop. disability service	1446	0.00	1548	0.04	1.03		1.07
doctor's room	1224	1.00	1497	1.00		1.22	1.22
private nursing practice	425	1.00	517	1.00		1.22	1.22
Other	5534	0.42	3753	0.34	0.76	0.56	0.68
Total	50489	0.28	55360	0.25	1.14	1.00	1.10
**RNs**							
hospital	25225	0.16	29195	0.19	1.11	1.40	1.16
nursing homes	5878	0.68	6225	0.48	1.70	0.75	1.06
community health	2914	0.00	4355	0.07	1.40		1.50
develop. disability service	1052	0.00	1116	0.04	1.02		1.06
doctor's room	1089	1.00	1329	1.00		1.22	1.22
private nursing practice	388	1.00	431	1.00		1.11	1.11
Other	4655	0.42	3170	0.34	0.77	0.55	0.68
Total	41201	0.28	45821	0.25	1.14	1.03	1.11
**Enrolled**							
hospital	5107	0.17	5621	0.17	1.11	1.04	1.10
nursing homes	2550	0.50	2260	0.39	1.10	0.68	0.89
community health	186	0.00	389	0.11	1.86		2.09
develop. disability service	394	0.00	432	0.04	1.06		1.10
doctor's room	135	1.00	168	1.00		1.24	1.24
private nursing practice	37	1.00	86	1.00		2.32	2.32
Other	879	0.38	583	0.34	0.71	0.59	0.66
Total	9288	0.29	9539	0.24	1.09	0.86	1.03

Notes: Observations with missing premises are excluded. RNs working as enrolled nurses are included in the enrolled. Nursing homes include hostels and hospices. The other category includes: schools, day procedures, various companies, prisons and the defense forces.

Table 2 shows that private sector hospitals have grown much faster than public sector hospitals (33% compared to 11%) however employment in private hospitals is still very small compared to the public sector institutions. The areas of rapid growth are the public sector nursing homes and community health. In the private sector, growth in private nursing practices and doctor's rooms has also been rapid.

Given the differences in employment growth across premises, one would have expected movements in retention probabilities over time. However, retention rates by age presented earlier suggest very stable retention probabilities over the analysis period. Are retention rates similar across premises? We turn to this question next.

Table 3 presents retention rates across three broad groups of premises for 1994–95 and 1999–2000. Retention rates are defined in the same manner as above; in particular the type of premises is that in which the nurse worked in the base year and nurses are treated as retained as long as they are working in the following year (in NSW) even if they have changed premises.

**Table 3:** Annual retention rates by sector and type of premises (%)

	**1994–95**			**1999–2000**			**99-94 differences**		
			
**Premises**	**Public**	**Private**	**Total**	**Public**	**Private**	**Total**	**Public**	**Private**	**Total**
**All nurses**									
hospital	83.8	81.9	83.5	84.6	83.9	84.5	0.8	2.0	1.0
nursing homes	80.9	83.5	82.5	84.4	85.7	85.0	3.6	2.2	2.5
all other premises	82.1	77.9	80.7	83.3	80.2	82.4	1.2	2.3	1.7
Total	83.2	81.3	82.7	84.3	83.4	84.1	1.1	2.1	1.4
**RNs**									
hospital	84.6	83.6	84.5	85.2	84.6	85.1	0.6	1.0	0.7
nursing homes	83.4	85.5	84.8	85.9	86.2	86.0	2.5	0.7	1.2
all other premises	83.3	79.2	81.9	84.2	81.6	83.5	0.9	2.4	1.6
Total	84.3	82.9	83.9	85.1	84.2	84.9	0.8	1.3	1.0
**Enrolled**									
hospital	79.5	74.0	78.6	81.3	79.5	81.0	1.8	5.5	2.5
nursing homes	76.9	76.8	76.9	81.0	83.8	82.0	4.0	7.0	5.1
all other premises	74.7	69.2	72.9	77.9	72.3	76.2	3.2	3.1	3.3
Total	78.2	74.4	77.1	80.7	79.3	80.4	2.5	4.9	3.3

Notes: The retention rate is conditional on working as a nurse in NSW in the base years (1994 or 1999). Nurses working in the base year are defined as retained if they are still employed as a nurse in NSW in the following year. Nursing homes include hostels and hospices.

Except for nursing homes, the retention rates in public sector premises are greater than in corresponding private premises. This holds for both time periods. Rates for nurses under 30 years of age show the same relative magnitudes in retention between public and private sectors. (Results are available from the authors.) The last set of columns in Table 3 shows the differences in retention rates over time. In all cases, these differences are positive; i.e. there are improvements in retention across all major groups of premises. The largest increases in retention are found in nursing homes and generally amongst enrolled nurses.

We do not find evidence of a problem with retention in nursing specific to public sector hospitals. Public sector hospitals have higher retention rates than other categories of premises except for private sector nursing homes. Furthermore, although the increase in retention in public hospitals has been slightly less compared to other premises, it has been positive. The stable aggregate retention rates shown earlier are a product of lessening differences in retention across sectors, and faster growth in the sectors with worse but more rapidly improving retention probabilities.

Retention in specific types of premises (i.e. the proportion of nurses who remain employed in the same sector rather than in nursing as a whole) show similar patterns (details available from the authors.) The average probability of remaining in the public hospital sector (for those already working in a public hospital) is 76% in 1994–95 and 77% in 1999–2000; corresponding figures for the private sector hospitals are 67% and 70% respectively.

Changes in predicted probabilities of retention are decomposed into components due to changes in coefficients and changes in the distribution of the characteristics. Table 4 summarizes the results. (Detailed results are available from the authors.) The main result from the decomposition is that there are only minor differences in either the distributions of the explanatory factors or in the coefficients. Very few sets of coefficients are statistically different over the two years and in particular the coefficients on type of premises are not jointly significantly different. The greater age and level of experience of nurses in 1999 did tend to increase retention rates but lower unemployment rates and shifts in the field away from clinical work caused a reduction in retention. The net effect is very small. Overall there has been very little change over time in retention levels and retention functions of personal and job characteristics.

**Table 4:** Decomposition of changes in retention rates over time

	**RNs**			**Enrolled**		
		
	**1999**	**1994**	**Differ**.	**1999**	**1994**	**Differ**.
Sample Size	45084	41386		9374	9095	
Observed mean retention probability	0.849	0.839	0.010	0.804	0.771	0.033
Predicted mean retention probability	0.849	0.839	0.010	0.804	0.771	0.033
Probability at mean characteristics:	0.857	0.848	0.009	0.813	0.781	0.031
Overall decomposition:						
Contribution from differences in coefficients			0.006			0.030
Contribution from differences in mean characteristics			0.004			0.001
Total			0.009			0.031
Contribution from individual characteristics:						
Personal characteristics (age, citizenship, sex)			0.002			0.012
Human capital (post basic qualification, years registered)			0.005			0.005
Location of job (region, unemployment rates)			-0.003			-0.003
Hours of work (hours in main job, second job as nurse)			0.003			-0.001
Job characteristics (classification, field, activity, premises)			-0.004			-0.012
Total			0.004			0.001
						
**Characteristics whose coefficients changed significantly between 1994 and 1999:**						
RNs: sex, hours in main job, job classification, field and activity.						
Enrolled: post-basic qualification and field						

Notes: Separate probits are estimated for 1999 and 1994 and for RNs and enrolled nurses. In both years the same explanatory variables are used and differences in the predicted retention probability is decomposed using a first order Taylor-series expansion.

### Transition probabilities across types of premises

We show in this section of the article that the stability in retention rates over time masks a substantial amount of movement by individuals from year to year across types of premises as well as in and out of nursing. We use the term 'churning' to describe this phenomenon. Using the long panel nature of the data we follow cohorts of nurses over time. In Table 5, four cohorts of RNs are chosen: a young cohort and a middle-aged cohort working in 1994 and cohorts in the same age groups working in 1997. In the top panel we follow the cohort of individuals who were working as RNs in 1994 and were aged 21–23 years at that time. This group numbers 1059. Most young nurses start their career in public sector hospitals and for the group represented in Table 5, we find that 70% were employed in public hospitals. One year later, 29.5% of the group are not working as nurses in NSW. Two years later the figure is 37%. The proportion not working then stays fairly stable at 37–43% up to year 2000 at which time the cohort is aged 27–29. If we follow the young cohort of working RNs in 1997 (the second panel in the table) we find even more concentration in the public hospitals. We also find a slightly increased proportion staying on as nurses (68 versus 63%); this is consistent with the results of improved retention presented above.

**Table 5:** Working history of cohorts of RNs, distribution by sector (%)

	**Cohort 1: 21 – 23 years old in 1994 (aged 27 – 29 in 2000)**						
	
**Sector**	**1994**	**1995**	**1996**	**1997**	**1998**	**1999**	**2000**
Public hospital	71	54	47	41		40	43
Private hospital	7	6	6	7		7	8
Public other	11	4	4	5		5	6
Private other	10	6	5	4		4	5
Missing premises	1	1	2	1		1	1
Not working		29	36	43		43	37
Total	100	100	100	100		100	100
No. observations	1059	1059	1059	1059		1059	1059
							
	**Cohort 2: 21 – 23 years old in 1997 (aged 24 – 26 in 2000)**						
	
**Sector**	**1997**	**1998**	**1999**	**2000**			
Public hospital	80		54	52			
Private hospital	11		8	8			
Public other	5		4	5			
Private other	3		2	2			
Missing premises	1		1	1			
Not working			31	32			
Total	100		100	100			
No. observations	1270		1270	1270			
							
	**Cohort 3: 41 – 43 years old in 1994 (aged 47 – 49 in 2000)**						
	
**Sector**	**1994**	**1995**	**1996**	**1997**	**1998**	**1999**	**2000**
Public hospital	47	40	38	39		37	36
Private hospital	9	9	9	8		8	8
Public other	24	19	19	21		21	21
Private other	19	17	14	12		11	12
Missing premises	1	3	4	2		2	2
Not working		12	16	18		21	21
Total	100	100	100	100		100	100
No. observations	4221	4221	4221	4221		4221	4221
							
	**Cohort 4: 41 – 43 years old in 1997 (aged 44 – 46 in 2000)**						
	
**Sector**	**1997**	**1998**	**1999**	**2000**			
Public hospital	49		42	40			
Private hospital	11		10	10			
Public other	22		21	21			
Private other	15		12	12			
Missing premises	3		2	2			
Not working			13	15			
Total	100		100	100			
No. observations	5232		5232	5232			

Notes: Four cohorts of working RNs in specified age groups and years are followed over time. The columns indicate the percentage distribution of the cohorts according to their work status and premises.

In comparison, we present similar results for the older cohort aged 41–43 in 1994. The proportion not working in the following year (13%) is much lower than for the younger age group reflecting the increased retention rates for older nurses, though this will include mature recent entrants to the workforce as well as experienced nurses. Again comparing to the same age cohort in 1997, we find a slightly improved retention rate at the end of the period. In some sense, Table 5 shows several of the results discussed previously, a stable profile of retention over time with a slight improvement in retention in nursing overall. Barring the youngest and oldest age groups, there is also a relatively stable retention profile over time by age cohorts.

In order to show in more detail the underlying flows, we look at the full matrix of transition probabilities across premises. Table 6 presents these figures for two of the cohorts described above; in each case we present the transition frequencies between 1999 and 2000. For example of the 452 RNs of cohort 1 who were working in public hospitals in 2000, 75% were also working in public hospitals in 1999, 2% were working in private hospitals, 1% were working in other public premises and 22% were not working. Of the 398 RNs not working in 2000, only 76% were not working in 1999.

**Table 6:** 1999–2000 transition frequencies for two cohorts of RNs (%)

	**Sector in 1999**							
	**Cohort 1: 21 – 23 years old in 1994 (aged 27 – 29 in 2000)**							
	
**Sector in 2000**	**No. obs**.	**Public hospital**	**Private hospital**	**Public other**	**Private other**	**Missing premises**	**Not working**	**Total**
Public hospital	452	75	2	1	0	0	22	100
Private hospital	83	10	62	1	4	0	23	100
Public other	65	12	3	52	8	2	23	100
Private other	50	12	4	4	56	4	20	100
Missing premises	11	18	18	0	10	18	36	100
Not working	398	15	3	3	2	1	76	100
Total	1059							
								
	**Cohort 3: 41 – 43 years old in 1997 (aged 44 – 47 in 2000)**							
	
**Sector in 2000**	**No. obs**.	**Public hospital**	**Private hospital**	**Public other**	**Private other**	**Missing premises**	**Not working**	**Total**
Public hospital	2088	89	2	2	1	0	6	100
Private hospital	510	8	78	3	4	1	6	100
Public other	1092	8	1	70	12	1	8	100
Private other	619	2	3	22	64	3	6	100
Missing premises	124	15	2	26	19	22	16	100
Not working	799	24	6	14	8	3	45	100
Total	5232							

Notes: These cohorts correspond to cohorts 1 and 3 from Table 5. The frequencies are in percentages and represent the distributions across the premises in 1999. For example, of the cohort 1 RNs working in public hospitals in 2000 (452 nurses), 75% were working in public hospitals in 1999, 22% were not working, 2% were working in private hospitals and so on.

The table shows that many RNs move between working and not working in any single year and a substantial number also move between sectors of employment. There is less movement for the older cohorts but it is still considerable. We find similar results for other years covered by the panel. It is possible that these movements are the result of dissatisfaction with the current job and represent a search for better working conditions. In this case policy intervention could not only improve nurses' job satisfaction and retention but also reduce possibly inefficient search and job switches across type of premises and out of the nursing workforce.

As an indication of the relative magnitude of movements between work and non-work and between type of premises for nursing, we calculate a mobility index from the transition information illustrated in Table 6. Using the Shorrocks [15] index of mobility, we find that mobility among the young cohort is 47% while the measure for older nurses is 45%. To give some idea of the importance of these values, Boeri and Flinn calculate values of 20% for occupational mobility among Italian women based on 9 occupational categories [16].

## Discussion

The profile of the NSW nursing workforce shows a substantial increase during the late 80s followed by a small trend upward in the 90s. The slow growth in the overall nursing workforce over the 90s can originate from a reduction in entry into nursing, a reduction in retention of nurses or an increasing tendency to retire early (reduced retention among higher age groups). It is important to recognise which of these forces is responsible for the relative slowdown in nursing employment as the recommended policy responses will differ. For example, increasing nurse intake from school leavers will have little impact if young nurses have high and increasing attrition rates. If increased attrition is concentrated in experienced nurses, then the appropriate policy response will focus on career paths and recognition for seniority and skill. Our evidence points to a problem in the entry of young nurses which is declining in the later years of the decade and which is too slow relative to demand.

Although retention is often discussed as an area of concern when discussing the nursing workforce, little work has been done on documenting its movements over time [1,9,10,17]. While the aging of the nursing workforce has received more attention, the relationship between aging and retention is rarely considered [2]. In general a reduction in retention while maintaining the age profile of new entrants will lead to a lowering of the average age of the workforce. However if this is accompanied by a reduction in entry, or an increase in the proportion of older entrants, then it will lead to an increase of the average age of the workforce.

The analysis presented above suggests that very little change has occurred over time in the retention rates in nursing. Furthermore, poor retention is not a problem particular to the public hospital sector. The finding of improved retention rates contradicts the widespread perception of a developing crisis in nursing retention. This implies that policies aimed at increasing the nursing positions in tertiary institutions may be more appropriate in the short term, as indeed is recommended by the National Review of Nursing Education [10].

This does not necessarily mean that there is no role for policy intervention in trying to improve retention. But further improvements in retention may require more focussed attention to identify specific aspects of working conditions. Shields and Ward found that although pay rates were important in determining overall job satisfaction, other aspects of the working environment were extremely important in explaining satisfaction and intentions to leave nursing [18]. The analysis of 'Magnet Hospitals' in the US carried out by Scott et al has shown that the attributes of nursing administration and leadership, nursing practice and nurse-doctor relationships are important in attracting and maintaining nursing staff [19]. Finally, Buchanan et al found that the most frequent reason for nurses working less was a change in family and/or personal life; hence more attention is needed to understand and help manage family and other demands [20].

In addition, churning in the younger age groups is an issue which is not well understood. As the nursing workforce is predominantly female, retention in nursing and job switching will be affected by child rearing responsibilities. What appears as an exit from the workforce in year to year transitions may in fact be a period spent caring for children followed by a return to the nursing workforce as children become more independent. Our results suggest that a substantial fraction of nurses who leave nursing return within a relatively short time frame (2 to 5 years). The administrative data unfortunately did not provide information on the nature of these periods away from nursing; in particular no information was available on whether the nurses were taking leave to sample other types of jobs or to raise children. As the National Nursing and Nursing Education Taskforce noted, there are many reasons why people change study, change jobs, and change careers but too few good studies that allow us to understand what drives these decisions [3].

## Conclusion

In this article, we investigate trends in the NSW nursing workforce over the the period 1993–2000 using administrative data collected by the NSW Nursing Registration Board and the Australian Institute of Health and Welfare. The dataset is longitudinal in that nurses can be linked across the years using a de-personalized identity number. This allows us to look at retention in nursing in NSW and to analyse retention probabilities by year, sector of work, age of the nurse and other personal and job characteristics.

Overall, several findings are obtained.

• Evidence of aging of the nursing workforce is found and this is caused by two reinforcing factors: a slower entry of young workers into the workforce and an increase in retention over time. The improved retention is found in most age groups, including the youngest group.

• Employment growth has been faster in the public sector overall but this is due to rapid increases in community health and nursing homes. When restricting attention to hospitals, we find that growth has been faster in the private sector.

• Retention is generally higher in public sector premises and is highest amongst nurses working in public hospitals.

• Retention is surprisingly stable over time. Probit regressions explaining retention show very little change in either the distributions of characteristics or in the estimated coefficients. The greater age and level of experience of nurses did tend to increase retention rates over time but lower unemployment rates and shifts in the nursing fields away from clinical work caused a reduction in retention. The net effect is very small.

• Net results based on stocks of nurses over time hide substantial flows from year to year across type of premises as well as in and out of employment in nursing. This is especially true for the younger age cohorts.

Job vacancies in the health care industry in Australia grew from 4,000 to 11,900 during the period 1993–2000. This suggests that there was excess demand in the sector as a whole and that the shortages reflected a problem of lack of supply. From our analysis, it follows that an appropriate focus is increasing entry into nursing but that encouraging retention of graduating nurses in the nursing workforce is also important.

Recent recommendations of the Productivity Commission addressing health workforce shortages stress the need for research to address recruitment, training, retention and re-entry, as well as exploring new roles for nurses and other health professionals and increasing flexibility in education and role definition [21]. Such research is also required to underpin further health service reforms including new models of service delivery and new methods of funding health services,. The data requirements to undertake comprehensive studies of nursing labour supply are substantial and we suggest that different types of data be collected: surveys of actual career choices by nurses as they move through and out of the nursing workforce and of stated preferences to explore the specific job factors which influence these decisions. Specialised questions on detailed working conditions are needed as well as questions regarding household-related decisions.

## Competing interests

The authors declare that they have no competing interests.

## Authors' contributions

DD and GJ designed the study and carried out the analyses. JH contributed to the conceptualisation and study design. All authors contributed to the development of the manuscript.
